# Editorial: Modern applications of EEG in neurological and cognitive research

**DOI:** 10.3389/fncom.2026.1969406

**Published:** 2026-09-10

**Authors:** Kuldeep Singh, Rajamanickam Yuvaraj, Geetanjali Sharma, Amit Joshi

**Affiliations:** 1 Department of Electronics Technology, Guru Nanak Dev University, Amritsar, Punjab, India; 2 Science of Learning in Education Centre, Office for Research, National Institute of Education, Nanyang Technological University, Singapore, Singapore; 3 Department of ECE, Maharaja Surajmal Institute of Technology, New Delhi, India; 4 Department of ECE, Malaviya National Institute of Technology, Jaipur, India

**Keywords:** biomarkers, brain-computer interface, cognitive neuroscience, electroencephalography (EEG), machine learning, neurological disorders, signal processing

Electroencephalography (EEG) remains one of the most accessible and temporally precise windows into human brain function. Since its first implementation nearly a century ago, EEG has evolved from a purely diagnostic tool into a versatile platform for the investigation of cognition, emotion, motor control, and disease across the lifespan. Recent advances in portable and low-density recording systems, signal processing, machine learning, and multimodal integration have greatly expanded the range of questions EEG can answer, from bedside diagnosis of neurodegenerative and neurological disorders, to naturalistic studies of learning, memory, and everyday behavior. To give a snapshot of the current state of the field, and to collect original contributions that demonstrate this growing scope, this Research Topic, “*Modern applications of EEG in neurological and cognitive research*” was created.

This call received a great response and the Research Topic includes 21 articles from authors around the globe across a range of article types including original research, brief research reports, perspectives and a correction. Below, we provide a brief overview of the accepted contributions, organized loosely by theme.

## EEG in diagnosis and clinical assessment

Several contributions addressed the use of EEG as a diagnostic or monitoring tool in clinical populations. Baron Shahaf et al. investigated whether a single-channel EEG marker is associated with perioperative cognitive change, suggesting simplified monitoring solutions for surgical settings. Jiang et al. characterized abnormal resting-state delta, theta and alpha oscillations, and dynamic functional connectivity in patients with mild cognitive impairment (MCI) using power spectral density and directed transfer function analyses, and reported frontoparietal delta enhancement, frontal alpha reduction, and correlations between connectivity strength and MoCA scores. Chriskos et al. used low-density, 8-channel EEG with functional connectivity and graph-theoretical features to classify subjects with primary progressive aphasia (PPA), MCI, and healthy controls, achieving perfect pairwise classification accuracy and demonstrating that short, low-cost recordings can support differential diagnosis of overlapping neurodegenerative syndromes. In the domain of epilepsy, Sharma et al. proposed iSeizdiag, a framework combining discrete wavelet transform-based feature extraction with machine learning classifiers for epileptic seizure detection. This work emphasizes the continued importance of EEG-based automated seizure diagnosis within the framework of smart healthcare applications. In the area of developmental and sensory disorders, Liang et al. compared early vs. late cochlear implantation in prelingually deaf children using a multi-feature auditory oddball paradigm. Their results showed that the P2, mismatch negativity (MMN), and late discriminative negativity (LDN) components together index delays in tone and vowel perception with later implantation age, with clear implications for the timing of clinical intervention.

## Memory, encoding, and retrieval

A series of papers focused on the electrophysiological correlates of memory processes. Vicentin et al. applied a hidden Markov model approach to decode the neural dynamics underlying everyday prospective remembering. Veliks et al. studied the effect of word list length on the event-related potential (ERP) and time-frequency signatures of episodic memory encoding and found a non-linear, list-length-dependent activation of language-processing brain areas. Zhang B. et al. used univariate ERP analysis combined with multivariate pattern analysis (MVPA) to explore the mechanisms underlying the production effect, the mnemonic advantage of reading words aloud compared to silently, in a mixed-list design, and found that this advantage is best explained by improved recollection/distinctiveness rather than familiarity/strength. A corrigendum to this article corrected an author affiliation, and this correction did not alter the scientific conclusions of the study. Zhou examined the effect of compound retrieval strategies within the framework of task switching.

## Attention, emotion, and affective processing

Other contributions dealt with the use of EEG as an index of attentional and emotional states. Liu and Zhang investigated the effects of high approach-motivated positive affect on selective attention under conditions of high perceptual load. Zhang H. et al. used steady-state visually evoked potentials (SSVEP) to investigate the modulation of evaluative valence on fear generalization in social anxiety. Ebato et al. compared relative usefulness of pupillometry, heart rate and EEG for multimodal fear detection. Reshad et al. carried out a pilot study where they explored the relationship between emotional states and the quality of the coding task, extending the EEG-based affect research to the field of applied, real-world productivity settings. Relatedly, Shao et al. combined quantitative emotional EEG with eye-tracking to evaluate the psychological healing potential of various street-design elements. This is an example of the growing role of EEG in environmental and architectural research.

## Stimulation, rehabilitation, and applied interventions

This Research Topic also featured work on EEG-guided or EEG-monitored interventions. Wang et al. explored the effects of amplitude-modulated transcranial alternating current stimulation (tACS) on working memory in college students. Miremadi et al. reported that virtual reality-based virtual embodiment training (VR-VET) is associated with relative alpha power modulation, providing candidate neuroimaging markers for future research in chronic pain rehabilitation.

## Methodology, reliability, and novel applications

Another set of papers addressed methodological issues of central importance to the future growth of the field. Korisky et al., assessed reliability of individual ERP level across different EEG recording systems, an important step toward precision EEG, and toward comparability of EEG recordings across laboratories. Chin et al. showed the feasibility of implementing a mobile EEG system to acquire resting-state and visual evoked potentials in young children in rural Ethiopia, highlighting the value of portable EEG for extending neuroscience studies to low-resource settings. Romo-De León et al. evaluated neurophysiological biometric patterns during semi-immersive compared to traditional learning experiences in the humanities to inform the design of immersive educational technologies. Finally, Morina wrote a forward-looking Perspective article, Leef, that proposed an environmental neuroscience modeling framework for cognitive resilience.

## Concluding remarks

The articles in this Research Topic illustrate the breadth of current EEG research, ranging from single-channel wearable markers and low-density diagnostic montages to multivariate decoding of memory and multimodal fusion with eye-tracking and stimulation techniques. They also emphasize common themes across the Research Topic including the search for more portable and accessible recording systems, the increasing sophistication of machine learning and multivariate analysis approaches applied to EEG signals, and the expansion of EEG research beyond the traditional clinic and laboratory to education, architecture, the workplace, and rehabilitation settings. We hope this Research Topic will serve as a useful reference and inspiration for researchers interested in applying EEG to novel questions in neurological and cognitive research, and we look forward to seeing how these themes develop in future work, including the second volume of this Research Topic series. We acknowledge the valuable contributions of all authors, the rigorous and constructive evaluations of the reviewers, and the support from the Frontiers editorial team throughout this process.

